# Supplementary material for: Adult male-specific inverse association between dry eye disease and intraocular pressure: KNHANES 2010–2012
Source: PLoS One. 2025 Feb 14;20(2):e0315010. doi: 10.1371/journal.pone.0315010 (PMC11828390; doi:10.1371/journal.pone.0315010)
Supplement: S1 Fig — IOP, intraocular pressure. (DOCX) [file pone.0315010.s001.docx]

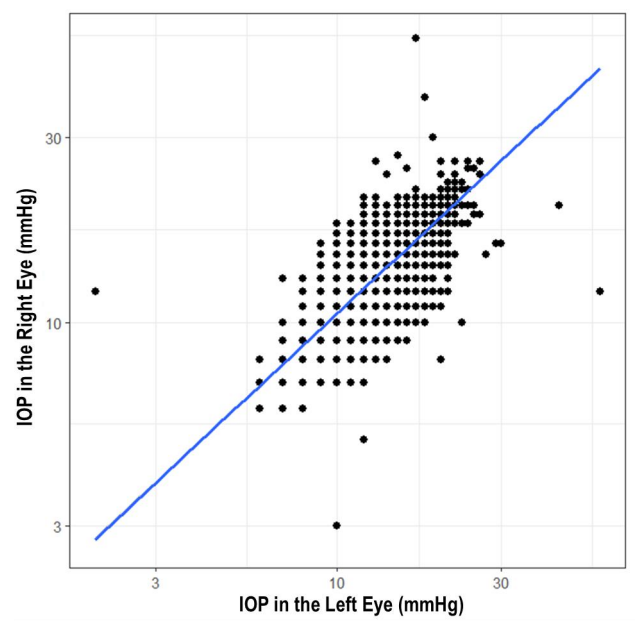


Figure S1. Scatter plot showing the correlation between IOP in the left and right eyes. IOP, intraocular pressure.
