# Supplementary material for: Adult male-specific inverse association between dry eye disease and intraocular pressure: KNHANES 2010–2012
Source: PLoS One. 2025 Feb 14;20(2):e0315010. doi: 10.1371/journal.pone.0315010 (PMC11828390; doi:10.1371/journal.pone.0315010)
Supplement: S1 Table — (DOCX) [file pone.0315010.s002.docx]

Table S1. Multiple linear regression analysis results for the effects of DED on IOP in the left eye (n = 13,194).

| **Variables** | **Total** | **Male** | **Female** |
| --- | --- | --- | --- |
|  | **β (95% CI)** | **β (95% CI)** | **β (95% CI)** |
| Model 1 |  |  |  |
| DED vs. no DED | 0.001 (-0.016, 0.015) | *-0.022 (-0.049, 0.004)* | 0.007 (-0.013, 0.027) |
| Model 2 |  |  |  |
| DED vs. no DED | 0.001 (-0.015, 0.017) | *-0.023 (-0.050, 0.004)* | 0.009 (-0.011, 0.029) |
| Model 3 |  |  |  |
| DED vs. no DED | *-0.025 (-0.052, 0.002)* | **-0.060 (-0.110, -0.011)** | -0.012 (-0.045, 0.021) |

CI, confidence interval; DED, dry eye disease

**Bold:** *p* < 0.05, *Italic*: *p* < 0.1

Model 1: adjustment for age, sex, survey year, region, income, and education

Model 2: model 1 + adjustment for alcohol drinking status, smoking status, exercise status, sleep duration, and body mass index

Model 3: model 2 + adjustment for family history of glaucoma, diabetes, and hypertension
