# Supplementary material for: Adult male-specific inverse association between dry eye disease and intraocular pressure: KNHANES 2010–2012
Source: PLoS One. 2025 Feb 14;20(2):e0315010. doi: 10.1371/journal.pone.0315010 (PMC11828390; doi:10.1371/journal.pone.0315010)
Supplement: S2 Table — (DOCX) [file pone.0315010.s003.docx]

Table S2. Multiple logistic regression analysis results for the effects of DED on high IOP (>21 mmHg) in the left eye (n = 13,194).

| **Variables** | **Total** | **Male** | **Female** |
| --- | --- | --- | --- |
|  | **OR (95% CI)** | **OR (95% CI)** | **OR (95% CI)** |
| Model 1 |  |  |  |
| DED vs. no DED | **0.40 (0.16, 0.99)** | **0.18 (0.04, 0.88)** | 0.53 (0.18, 1.56) |
| Model 2 |  |  |  |
| DED vs. no DED | *0.42 (0.17, 1.04)* | **0.18 (0.04, 0.89)** | 0.55 (0.19, 1.59) |
| Model 3 |  |  |  |
| DED vs. no DED | *0.43 (0.17, 1.06)* | **0.18 (0.04, 0.91)** | 0.57 (0.20, 1.65) |

CI, confidence interval; DED, dry eye disease; OR, odds ratio

**Bold:** *p* < 0.05, *Italic*: *p* < 0.1

Model 1: adjustment for age, sex, survey year, region, income, and education

Model 2: model 1 + adjustment for alcohol drinking status, smoking status, exercise status, sleep duration, and body mass index

Model 3: model 2 + adjustment for family history of glaucoma, diabetes, and hypertension
