# Supplementary material for: Adult male-specific inverse association between dry eye disease and intraocular pressure: KNHANES 2010–2012
Source: PLoS One. 2025 Feb 14;20(2):e0315010. doi: 10.1371/journal.pone.0315010 (PMC11828390; doi:10.1371/journal.pone.0315010)
Supplement: S3 Table — (DOCX) [file pone.0315010.s004.docx]

Table S3. Multiple linear regression analysis results for the effects of DED on IOP after excluding sampling weights (n = 13,194).

| **Variables** | **Total** | **Male** | **Female** |
| --- | --- | --- | --- |
|  | **β (95% CI)** | **β (95% CI)** | **β (95% CI)** |
| Model 1 |  |  |  |
| DED vs. no DED | -0.001 (-0.012, 0.010) | *-0.021 (-0.044, 0.003)* | 0.005 (-0.008, 0.017) |
| Model 2 |  |  |  |
| DED vs. no DED | -0.001 (-0.011, 0.011) | *-0.020 (-0.044, 0.003)* | 0.006 (-0.007, 0.019) |
| Model 3 |  |  |  |
| DED vs. no DED | **-0.023 (-0.046, -0.001)** | **-0.055 (-0.104, -0.006)** | -0.018 (-0.044, 0.008) |

CI, confidence interval; DED, dry eye disease

**Bold:** *p* < 0.05, *Italic*: *p* < 0.1

Model 1: adjustment for age, sex, survey year, region, income, and education

Model 2: model 1 + adjustment for alcohol drinking status, smoking status, exercise status, sleep duration, and body mass index

Model 3: model 2 + adjustment for family history of glaucoma, diabetes, and hypertension
