# Supplementary material for: Adult male-specific inverse association between dry eye disease and intraocular pressure: KNHANES 2010–2012
Source: PLoS One. 2025 Feb 14;20(2):e0315010. doi: 10.1371/journal.pone.0315010 (PMC11828390; doi:10.1371/journal.pone.0315010)
Supplement: S4 Table — (DOCX) [file pone.0315010.s005.docx]

Table S4. Multiple logistic regression analysis results for the effects of DED on high IOP (>21 mmHg) after excluding sampling weights (n = 13,194).

| **Variables** | **Total** | **Male** | **Female** |
| --- | --- | --- | --- |
|  | **OR (95% CI)** | **OR (95% CI)** | **OR (95% CI)** |
| Model 1 |  |  |  |
| DED vs. no DED | **0.19 (0.18, 0.20)** | **0.32 (0.30, 0.34)** | **0.12 (0.11, 0.13)** |
| Model 2 |  |  |  |
| DED vs. no DED | **0.21 (0.20, 0.22)** | **0.27 (0.25, 0.28)** | **0.17 (0.16, 0.18)** |
| Model 3 |  |  |  |
| DED vs. no DED | **0.20 (0.19, 0.21)** | **0.25 (0.23, 0.26)** | **0.18 (0.16, 0.19)** |

CI, confidence interval; DED, dry eye disease; OR, odds ratio

**Bold:** *p* < 0.05

Model 1: adjustment for age, sex, survey year, region, income, and education

Model 2: model 1 + adjustment for alcohol drinking status, smoking status, exercise status, sleep duration, and body mass index

Model 3: model 2 + adjustment for family history of glaucoma, diabetes, and hypertension
