# Supplementary material for: Adult male-specific inverse association between dry eye disease and intraocular pressure: KNHANES 2010–2012
Source: PLoS One. 2025 Feb 14;20(2):e0315010. doi: 10.1371/journal.pone.0315010 (PMC11828390; doi:10.1371/journal.pone.0315010)
Supplement: S5 Table — (DOCX) [file pone.0315010.s006.docx]

Table S5. Multiple linear regression analysis results for the effects of DED on IOP after incorporating multiple imputations (n = 15,043).

| **Variables** | **Total** | **Male** | **Female** |
| --- | --- | --- | --- |
|  | **β (95% CI)** | **β (95% CI)** | **β (95% CI)** |
| Model 1 |  |  |  |
| DED vs. no DED | -0.005 (-0.020, 0.011) | -0.020 (-0.046, 0.006) | -0.001 (-0.019, 0.018) |
| Model 2 |  |  |  |
| DED vs. no DED | -0.003 (-0.019, 0.012) | -0.020 (-0.046, 0.007) | 0.001 (-0.017, 0.020) |
| Model 3 |  |  |  |
| DED vs. no DED | -0.002 (-0.016, 0.013) | **-0.024 (-0.047, -0.001)** | 0.001 (-0.017, 0.020) |

CI, confidence interval; DED, dry eye disease

**Bold:** *p* < 0.05

Model 1: adjustment for age, sex, survey year, region, income, and education

Model 2: model 1 + adjustment for alcohol drinking status, smoking status, exercise status, sleep duration, and body mass index

Model 3: model 2 + adjustment for family history of glaucoma, diabetes, and hypertension
