# Supplementary material for: Adult male-specific inverse association between dry eye disease and intraocular pressure: KNHANES 2010–2012
Source: PLoS One. 2025 Feb 14;20(2):e0315010. doi: 10.1371/journal.pone.0315010 (PMC11828390; doi:10.1371/journal.pone.0315010)
Supplement: S6 Table — (DOCX) [file pone.0315010.s007.docx]

Table S6. Multiple logistic regression analysis results for the effects of DED on high IOP (>21 mmHg) after incorporating multiple imputations (n = 15,043).

| **Variables** | **Total** | **Male** | **Female** |
| --- | --- | --- | --- |
|  | **OR (95% CI)** | **OR (95% CI)** | **OR (95% CI)** |
| Model 1 |  |  |  |
| DED vs. no DED | *0.44 (0.17, 1.15)* | *0.30 (0.08, 1.04)* | 0.54 (0.14, 2.05) |
| Model 2 |  |  |  |
| DED vs. no DED | *0.40 (0.15, 1.05)* | *0.26 (0.07, 1.01)* | 0.58 (0.15, 2.20) |
| Model 3 |  |  |  |
| DED vs. no DED | **0.34 (0.12, 0.95)** | **0.27 (0.07, 0.99)** | 0.59 (0.15, 2.25) |

CI, confidence interval; DED, dry eye disease; OR, odds ratio

**Bold:** *p* < 0.05, *Italic*: *p* < 0.1

Model 1: adjustment for age, sex, survey year, region, income, and education

Model 2: model 1 + adjustment for alcohol drinking status, smoking status, exercise status, sleep duration, and body mass index

Model 3: model 2 + adjustment for family history of glaucoma, diabetes, and hypertension
