# Supplementary material for: Adult male-specific inverse association between dry eye disease and intraocular pressure: KNHANES 2010–2012
Source: PLoS One. 2025 Feb 14;20(2):e0315010. doi: 10.1371/journal.pone.0315010 (PMC11828390; doi:10.1371/journal.pone.0315010)
Supplement: Attachment — Submitted filename: Response to Reviewers.docx [file pone.0315010.s008.docx]

**Response to Comments (Manuscript ID PONE-D-24-25756)**

**Adult male-specific inverse association between dry eye disease and intraocular pressure: KNHANES 2010–2012**

Yun-Hee Choi, Martha Kim, Yoon-Hyeong Choi^*^, Dong Hyun Kim^*^

**Journal Requirements:**

| *Comment 1* | *1. When submitting your revision, we need you to address these additional requirements.*  *Please ensure that your manuscript meets PLOS ONE's style requirements, including those for file naming. The PLOS ONE style templates can be found at*  *https://journals.plos.org/plosone/s/file?id=wjVg/PLOSOne_formatting_sample_main_body.pdf and*  *https://journals.plos.org/plosone/s/file?id=ba62/PLOSOne_formatting_sample_title_authors_affiliations.pdf* |
| --- | --- |
| Response 1 | We have confirmed that our manuscript meets PLOS ONS’s style requirements. Please see the revised manuscript. |
| *Comment 2* | *2. Your ethics statement should only appear in the Methods section of your manuscript. If your ethics statement is written in any section besides the Methods, please move it to the Methods section and delete it from any other section. Please ensure that your ethics statement is included in your manuscript, as the ethics statement entered into the online submission form will not be published alongside your manuscript.* |
| Response 2 | We have revised that ethics statement should only appear in the Methods section. Please see the revised manuscript. |
| *Comment 3* | *3. Please review your reference list to ensure that it is complete and correct. If you have cited papers that have been retracted, please include the rationale for doing so in the manuscript text, or remove these references and replace them with relevant current references. Any changes to the reference list should be mentioned in the rebuttal letter that accompanies your revised manuscript. If you need to cite a retracted article, indicate the article’s retracted status in the References list and also include a citation and full reference for the retraction notice.* |
| Response 3 | We have revised all our reference list complete and correct. There are no cited papers that have been retracted. Also, any changes to the reference list are described in the revision letter. |

**Additional Editor Comments:**

| *General*  *Comment* | *It interests me that your study demonstrates a significant negative association between DED and IOP in Korean males using a large-scale general population epidemiological study. However, overall, there are several areas for improvement. For improvement, in addition to what I mentioned, please respond well to the points discussed below by two reviewers.* |
| --- | --- |
| Response | We deeply appreciate the constructive comments provided by the editor. We appreciate the editor’s constructive, detailed comments which helped us further improve our manuscript. Below we address your comments and list of changes that we made to our manuscript according to your reports. We believe that these modifications have strengthened the manuscript. We hope that the revised manuscript is suitable for publication in the *PLOS ONE*. |
| *Comment 1* | *[Major comments]*  *1.In Table 1, it is noticeable that the percentage of DED cases among all subjects is 7.8% overall and 8.6% in urban areas, which appears to be significantly lower compared to the prevalence of DED in Asian countries in DEWSII epidemiology report. This is likely due to the criteria requiring both DED symptoms and a doctor's diagnosis. However, this may result in distortions due to patients' tendencies regarding hospital visits (i.e., whether they visit a hospital with the same degree of DED). This should be included as a limitation in the discussion.* |
| Response 1 | We thank the editor for pointing out where additional clarity is needed. The reviewer is right. As we used both DED symptoms and doctor’s diagnosis to define DED, patient’s tendencies regarding hospital visits can result distortions. Based on the reviewer’s advice, we have revised the manuscript. Please see lines 368–370 in the revised manuscript.  **Line 368:** “Moreover, patients’ willingness to seek medical attention, despite experiencing similar symptoms, may lead to misclassification of DED.” |
| *Comment 2* | *2.In Table 2, there is no relationship between IOP and DED in Models 1 and 2, but there is a significant relationship in Model 3. The difference between Models 2 and 3 lies in the inclusion of family history of glaucoma, diabetes, and hypertension. It would be beneficial to add a detailed analysis of the significance of these differences between the models in the discussion. Additionally, it would be helpful to discuss the necessity of such a complex analysis system and explain the criteria used to classify risk factors into Models 1 to 3.* |
| Response 2 | We thank the editor for pointing out where additional clarity is needed. Based on the editor’s advice, we have added an explanation that, to examine the impact of different types of covariates on the results, we conducted analyses by dividing the covariates into following three distinct groups: 1) demographic variables, 2) anthropometric measurements and health-related behaviors, and 3) genetic or disease variables, to assess their influence on the outcome. Please see lines 163–166 and in the revised manuscript.  **Line 163:** “We developed three sequential models, differentiated by (1) demographic variables, (2) anthropometric measurements and health-related behaviors, and (3) genetic or disease variables, to assess their influence on the outcome [34].”  **Reference:** “Choi, Y. H., Huh, D. A., & Moon, K. W. (2021). Joint Effect of Alcohol drinking and environmental cadmium exposure on hypertension in Korean adults: analysis of data from the Korea National Health and Nutrition Examination Survey, 2008 to 2013. *Alcoholism: Clinical and Experimental Research*, *45*(3), 548-560.”  The following references were additionally added.  Choi, Y. H., Huh, D. A., & Moon, K. W. (2021). Joint Effect of Alcohol drinking and environmental cadmium exposure on hypertension in Korean adults: analysis of data from the Korea National Health and Nutrition Examination Survey, 2008 to 2013. *Alcoholism: Clinical and Experimental Research*, *45*(3), 548-560.  Moreover, we have added a discussion in the interpretation section addressing why the inverse association between DED and IOP did not show significant effects in models 1 and 2, but emerged in model 3. Please see lines 341–349 in the revised manuscript.  **Line 341:** “As evidenced by multiple covariate adjustment models and subgroup analyses, the effect of DED on IOP varied according to demographic and disease-related characteristics. The inverse association between DED and IOP was not significant in models 1 and 2 (table 2), which adjusted for demographic characteristics and health-related behaviors. However, a significant association was observed in model 3, which additionally adjusted for genetic and disease-related factors, suggesting that family history of glaucoma, diabetes, and hypertension play crucial roles in this relationship. This finding was further elucidated in the subgroup analyses, which revealed a more pronounced inverse association between DED and IOP among males with higher household income and education levels who did not have hypertension.” |
| *Comment 3* | *3.Regarding the sudden change in the percentage of DED cases by survey year in Table 1 (7.1% in 2010, 6.3% in 2011, and a sharp increase to 10.3% in 2012), how can this be explained? Such a drastic change could raise questions about the reliability of the data used in this analysis, so a possible explanation or acknowledgment of limitations is needed. Considering that only 55 out of 19,599 patients were undergoing glaucoma treatment, could this be considered too few, given the general prevalence of glaucoma in Korea? The use of glaucoma medication could be a significant variable in the results. Is there a possibility of insincere responses?* |
| Response 3 | We thank the editor for the opportunity to further clarify these points. Although our study, which excluded some participants, observed an increase in DED prevalence from 6.3–7.1% in 2010–2011 to 10.3% in 2012, these findings closely align with another KNHANES 2010–2011 and 2012 study that reported physician-diagnosed DED prevalence of 8% and 10.4%, respectively, among the general adult population [Ahn et al., 2014; Kim et al., 2016]. Therefore, while our observed DED prevalence may be lower than typical estimates due to our use of both diagnostic and symptomatic criteria, it can be considered representative of the DED prevalence observed in Korean adults.  Meanwhile, the increasing prevalence of DED over this period is primarily attributed to the rapid proliferation and use of smartphones and the rise in air pollution during this time period. We have incorporated these explanations into our discussion and limitations sections. Please see lines 277–281 and 366–368 in the revised manuscript.  **Line 277:** “This study utilized data from the KNHNAES (2010–2012), which represents the general population of South Korea, identified that the prevalence of DED increased from 7.1% to 10.3% during this period. This figure is similar to the 8% to 10.4% prevalence reported in national statistics for the same period [36,37], which is likely influenced by air pollution levels and the widespread use of smartphones [36,37].”  **Line 366:** “Second, DED was ascertained based on both self-reported symptoms and physician diagnoses. Therefore, DED prevalence reported in this study may differ from the actual prevalence in the Korean population [36,37].”  **Reference:** “Ahn, J. M., Lee, S. H., Rim, T. H. T., Park, R. J., Yang, H. S., Im Kim, T., ... & Society, O. (2014). Prevalence of and risk factors associated with dry eye: the Korea National Health and Nutrition Examination Survey 2010–2011. *American journal of ophthalmology*, *158*(6), 1205-1214.  Kim, M., Oh, J. H., Park, C. Y., & Lee, S. W. (2016). Dry eye disease and allergic conditions: a Korean nationwide population-based study. *American journal of rhinology & allergy*, *30*(6), 397-401.”  The following references were additionally added.  Ahn, J. M., Lee, S. H., Rim, T. H. T., Park, R. J., Yang, H. S., Im Kim, T., ... & Society, O. (2014). Prevalence of and risk factors associated with dry eye: the Korea National Health and Nutrition Examination Survey 2010–2011. *American journal of ophthalmology*, *158*(6), 1205-1214.  Kim, M., Oh, J. H., Park, C. Y., & Lee, S. W. (2016). Dry eye disease and allergic conditions: a Korean nationwide population-based study. *American journal of rhinology & allergy*, *30*(6), 397-401.  Furthermore, it is important to note that the 55 individuals excluded due to ongoing glaucoma treatment do not represent the total number of patients undergoing glaucoma treatment in the general population. This subset was identified after sequentially excluding those without DED and IOP measurements (2,880 individuals) and those who had undergone cataract or refractive surgery (1,621 individuals) from the initial 19,599 participants. In accordance with the editor's suggestion and our own assessment that glaucoma medication could significantly impact the results, we chose to exclude these subjects from our analysis.  Lastly, given that the KNHANES is conducted with researchers explaining the questionnaire to participants, the likelihood of insincere responses is minimal. Even if such responses exist, they are likely too few to substantially influence the results. |
| *Comment 4* | *[Minor comments]*  *4. Lines 74–76: Did they really report that the non-glaucoma group with DED had significantly lower IOP than the non-glaucoma group without DED? Check it out. (PMID: 32811862)* |
| Response 4 | We thank the editor for the opportunity to further clarify this point. The authors report that the non-glaucoma group with DED had lower IOP than the non-glaucoma group without DED during spring to fall; however, they did not conduct statistical analysis to assess those relationships. As our previous descriptions were unclear, we have revised the sentences to better highlight statistically significant relationships between IOP and DED-related indicators. Please see lines 64–68 in the revised manuscript.  **Line 64:** “One study found a significant inverse correlation between IOP and corneal staining score, which showed higher values in DED patients, regardless of their glaucoma status [22]. Another study showed that IOP was significantly lower in patients with a short tear break-up time (TBUT), a characteristic of DED [23].” |
| *Comment 5* | *5. It needs to be clearly stated whether the results of the multiple regression are beta values or odds ratios.* |
| Response 5 | We thank the editor for the opportunity to further clarify these points. The results of multiple regression are beta values. Based on the reviewer’s advice, we have further clarified this point in the statistical analyses section. Please see lines 157–159 in the revised manuscript. **Line 157:** “DED was categorized as a binary variable (presence or absence), while IOP was treated as a continuous variable. The association was assessed by comparing the effects of DED on IOP level and estimating regression coefficients (β) and 95% confidence intervals (CIs).” Moreover, we have clarified the that the results of multiple linear regression analysis were β (95% CI) in Table 2. Please see the table 2 in the revised manuscript. **Table 2:** “Multiple linear regression analysis results for the effects of dry eye disease on intraocular pressure (n = 13,194).” **Variables** \| **Total** \| **Male** \| **Female**; \| **β (95% CI)** \| **β (95% CI)** \| **β (95% CI)**; Model 1 \| \| \|; DED vs. no DED \| -0.005 (-0.021, 0.011) \| -0.023 (-0.051, 0.005) \| 0.001 (-0.019, 0.021); Model 2 \| \| \|; DED vs. no DED \| -0.003 (-0.019, 0.0124) \| *-0.024 (-0.052, 0.005)* \| 0.003 (-0.017, 0.023); Model 3 \| \| \|; DED vs. no DED \| **-0.032 (-0.059, -0.004)** \| **-0.059 (-0.106, -0.012)** \| -0.020 (-0.055, 0.015); Model 1 \| \| \| **Bold:** *p* < 0.05, *Italic*: *p* < 0.1 Model 1: adjustment for age, sex, survey year, region, income, and education Model 2: model 1 + adjustment for alcohol drinking status, smoking status, exercise status, sleep duration, and body mass index Model 3: model 2 + adjustment for family history of glaucoma, diabetes, and hypertension Abbreviations: CI, confidence interval; DED, dry eye disease |
| *Comment 6* | *6. Tables S5 and S7 show positive values, unlike the other tables. In the text, there is a mention of "lower IOP," yet the title is set as "high IOP." It is necessary to check whether the descriptions, titles, and figures in these tables are accurate and consistent with the other tables.* |
| Response 6 | We thank the editor for the opportunity to check the descriptions of Table S5 and S7. The results presented in Tables S5 and S7 are derived from multiple logistic regression analyses, with odds ratios (ORs) as the primary measure. These ORs should be interpreted as indicating a negative association when less than 1 and a positive association when greater than 1. Consequently, the significant values shown in Model 3 of Tables S5 and S7 suggest that individuals with DED have a lower likelihood of experiencing high IOP (IOP) (>21 mmHg) compared to those without DED. We have revised the wording to avoid potential confusion arising from the term "Lower IOP." Additionally, Tables S5 and S7 have been renumbered to S4 and S6, respectively. Please see lines 268–272 in the revised manuscript.  **Line 268:** “According to the sensitivity analysis results of accounting for the left eye, DED and IOP showed inverse associations in the overall population along with males in the fully adjusted model (model 3; S1-S2 Tables). Even in models without complex sample weights, males with DED showed a significant inverse association with IOP in the fully adjusted model (model 3; S3-S4 Tables).” |
| *Comment 7* | *7. The criteria for high IOP are defined as ">21" in S2 and "≥18" in S3, but there are no criteria listed for Tables 2, S5, and S7. Although line 125 of the paper states that high IOP is defined as">21," why is the criterion of"≥18" used in Table S3? This is very confusing.* |
| Response 7 | We thank the editor for the opportunity to correct this point. Initially, we aimed to verify whether our results would hold using an alternative definition of high intraocular pressure (IOP) at ≥18 mmHg. However, we failed to adequately explain this in the manuscript, which may have led to misunderstandings. To align with our original objective of confirming the results for high IOP (>21 mmHg) under various conditions - using left eye data, exclusion of sample weights, and adding missing data of covariates using multiple imputation - we have eliminated Table S3 and adjusted the table order accordingly. Furthermore, we have clearly specified in both the main text and supplementary data that all logistic regression results pertain to high IOP (>21 mmHg). Please see lines 268–274 in the revised manuscript.  **Line 268:** “According to the sensitivity analysis results of accounting for the left eye, DED and IOP showed inverse associations in the overall population along with males in the fully adjusted model (model 3; S1-S2 Tables). Even in models without complex sample weights, males with DED showed a significant inverse association with IOP in the fully adjusted model (model 3; S3-S4 Tables). In the analysis in which the missing values had been imputed using multiple imputation methods for participants without covariates, significant results observed in the main analysis persisted in the fully adjusted model (model 3; S5-S6 Tables).” |
| *Comment 8* | *8. Figure 2: The survey year and region, which were included in Model 1, are omitted. Please add them.* |
| Response 8 | We thank the editor for pointing out where additionally clarity is needed. Initially, we conducted analyses only on specific subgroups that we deemed significant. However, in response to the editor's suggestions, we expanded our analysis to include subgroup analyses for all covariates. As a result, we discovered significant subgroup differences by region in the total population, and by household income and education level among males. Consequently, we have revised our results to reflect these new findings. Please see lines 170–176, figure 2, 251–260, and 346–361 in the revised manuscript.  **Line 170:** “We also conducted fully adjusted associations between DED and IOP in subgroups stratified by age (20–39 or ≥40 years), survey year (2010, 2011, or 2012), region (urban or rural), household income (low, low to medium, medium to high, or high), education level (below high school, high school, or college or higher), alcohol consumption (never, former, or current), smoking status (never, former, or current), exercise status (vigorous, moderate, or little), sleep duration (<7 or ≥7 h), BMI (<25 or ≥25 kg/m^2^), family history of glaucoma (no or yes), diabetes (no or yes), and hypertension (no or yes).”    **Fig 2.** **Association between DED and IOP among subgroups. Black asterisks (*) indicate significant associations in each group (*p* < 0.05).** Red asterisks (*) indicate significant differences between the groups (*p*-interaction < 0.05). Abbreviations: BMI, body mass index; DED, dry eye disease; IOP, intraocular pressure; 95% CI, 95% confidence interval.  **Line 251:** “In the overall population, participants residing in urban areas exhibited a significantly stronger inverse association between DED and IOP compared to their rural counterparts (urban: -0.051 [-0.086, -0.016] vs. rural: 0.026 [-0.019, 0.072]; *p*-interaction < 0.05). Among males, the inverse association was significantly stronger in participants with more than medium level of household income (high: -0.076 [-0.144, -0.007] vs medium to high: -0.175 [-0.262, -0.087] vs low to medium: 0.001 [-0.098, 0.100] vs low: 0.042 [-0.042, 0.126]), college or higher level of education (college or higher: -0.101 [-0.164, -0.038] vs high school: -0.032 [-0.213, 0.148] vs below high school: -0.03 [-0.104, 0.044]), and those without hypertension (no: -0.083 [-0.136, -0.031] vs. yes: 0.003 [-0.090, 0.096]) (all *p*-interaction < 0.05).”  **Line 346:** “This finding was further elucidated in subgroup analyses, which revealed a more pronounced inverse association between DED and IOP among males with higher household income and education levels who did not have hypertension. Low household income and education level were well-established risk factors for glaucoma [58]. Additionally, increase in systolic blood pressure and heart rate influences in rise in IOP [59]. Although the differences between groups were not significant, a negative association between DED and IOP was observed among males who were young adults (20–39 years), urban residents, non-drinkers, non-smokers, slept more than 7 hours, and those without diabetes. It is known that age over 40 years, alcohol consumption, smoking, sleep deprivation, and diabetes are considered risk factors for elevated IOP and glaucoma. These factors contribute to the risk through various mechanisms, including osmotic effects [60], vascular problems [16,61], and alterations in sleep hormones [30] or aqueous outflow [62]. Therefore, the results of this study indicate that different treatment strategies for glaucoma management should be considered for males with lower household income, education levels, or hypertension, along with consideration of their lifestyle factors.”  **Reference:** “Ko, Y. C., Hwang, D. K., Chen, W. T., Lee, C. C., & Liu, C. J. (2016). Impact of socioeconomic status on the diagnosis of primary open-angle glaucoma and primary angle closure glaucoma: a nationwide population-based study in Taiwan. *PloS one*, *11*(2), e0149698.”  The following references were additionally added.  Ko, Y. C., Hwang, D. K., Chen, W. T., Lee, C. C., & Liu, C. J. (2016). Impact of socioeconomic status on the diagnosis of primary open-angle glaucoma and primary angle closure glaucoma: a nationwide population-based study in Taiwan. *PloS one*, *11*(2), e0149698. |
| *Comment 9* | *9. Lines 251–252: Shouldn't "significantly lower" be corrected to "significantly stronger negative correlation"? The meaning could be interpreted entirely oppositely.* |
| Response 9 | We thank the editor for the opportunity to correct the interpretations. Initially, we aimed to demonstrate that the inverse association between DED and IOP was observed in certain groups. As the statistical differences between groups were not always significant (*p*-interaction), we were unable to conclusively state that specific groups displayed a more pronounced inverse relationship between DED and IOP compared to others. Based on the editor’s advice, we have revised our figure and presented only the results that showed statistically significant differences between groups. Please see lines 251–260 in the revised manuscript.  **Line 251:** “In the overall population, participants residing in urban areas exhibited a significantly stronger inverse association between DED and IOP compared to their rural counterparts (urban: -0.051 [-0.086, -0.016] vs. rural: 0.026 [-0.019, 0.072]; *p*-interaction < 0.05). Among males, the inverse association was significantly stronger in participants with more than medium level of household income (high: -0.076 [-0.144, -0.007] vs medium to high: -0.175 [-0.262, -0.087] vs low to medium: 0.001 [-0.098, 0.100] vs low: 0.042 [-0.042, 0.126]), college or higher level of education (college or higher: -0.101 [-0.164, -0.038] vs high school: -0.032 [-0.213, 0.148] vs below high school: -0.03 [-0.104, 0.044]), and those without hypertension (no: -0.083 [-0.136, -0.031] vs. yes: 0.003 [-0.090, 0.096]) (all *p*-interaction < 0.05).” |
| *Comment 10* | *10. Line 295: The expression "in vitro mouse study" seems odd. Please check the expression.* |
| Response 10 | We thank the editor for the opportunity to correct these points. Based on the reviewer’s advice, we have revised the expression “*in vitro* mouse study” to “mouse study”. Please see line 301 in the revised manuscript.  **Line 301:** “In a mouse study, the PGE2 or PGF2α concentration in tears significantly increased among patients with DED compared to those without DED.” |
| *Comment 11* | *11. Lines 337–339: Alcohol leads to a reduction in the production of aqueous humor, which lowers IOP, so this is the opposite mechanism of increasing IOP by Alcohol. A more appropriate reason should be found.* |
| Response 11 | We thank the editor for the opportunity to correct these points. The reviewer is right. We revised the explanation of the mechanism by which alcohol increases IOP. Given that our previous subgroup analysis did not show significant differences between groups regarding alcohol consumption, we opted to present a simplified description of the mechanism. Please see lines 352–358 in the revised manuscript.  **Line 352:** “Although the differences between groups were not significant, a negative association between DED and IOP was observed among males who were young adults (20–39 years), urban residents, non-drinkers, non-smokers, slept more than 7 hours, and those without diabetes. It is known that age over 40 years, alcohol consumption, smoking, sleep deprivation, and diabetes are considered risk factors for elevated IOP and glaucoma. These factors contribute to the risk through various mechanisms, including osmotic effects [60], vascular problems [16,61], and alterations in sleep hormones [30] or aqueous outflow [62].” |

**Reviewer 1**

| *General*  *Comment* | *Reviewer #1: The relevance of reviewed article beyond doubt due to the prevalence of advanced dry eye syndrome. The study of the relationship of dry eye syndrome with various general and ophthalmological parameters (in particular, IOP) is of great interest for attention.*  *The article is presented in an accessible form and in understandable language. The abstract fully reflects the material presented. The tables are clear. The conclusions correspond to the stated goals.*  *The statistical methods that were used at each stage of the population study are sufficiently described. This proves the significance of the data analysis results.*  *During the review of the results, there were some questions that required clarification:* |
| --- | --- |
| Response | We thank the reviewer for the time and effort to review our manuscript as well as their encouraging comments. Below we address your comments and list of changes that we made to our manuscript according to your reports. We believe that these modifications have strengthened the manuscript. We hope that the revised manuscript is suitable for publication in the *PLOS ONE*. |
| *Comment 1* | *In the “Definition of DED” section of the “Materials and Methods” chapter, it would be worthwhile to describe in more detail which questionnaire is used to determine the definition of DED.* |
| Response 1 | We thank the reviewer for the opportunity to further clarify these points. Based on the reviewer’s advice, we have added more information regarding the determination of the DED definition. Please see lines 106–112 in the revised manuscript.  **Line 106:** “Assessment of DED was conducted using a self-reported questionnaire among aged 19 years and older. The DED questionnaire items were constructed based on a standardized guideline developed by the Korean Ophthalmological Society for the assessment of ocular health among the Korean population [26]. The following two questionnaire items were used to assess DED: “Have you ever been diagnosed with DED by a medical doctor?” and “Have you ever experienced dry eye symptoms such as dryness or irritation in your eyes, excluding cases where symptoms occurred occasionally or intermittently?”.”  **Reference:** “Korean Ophthalmological Society. Quality Control and Standardization of Indicators for Ophthalmic Examination Specialists (Ophthalmology Residents) in the First Year (2010) of the Fifth Korea National Health and Nutrition Examination Survey. Osong, Cheongju, Korea: Korea Centers for Disease and Control and Prevention. 2011.”  The following references were additionally added.  Korean Ophthalmological Society. Quality Control and Standardization of Indicators for Ophthalmic Examination Specialists (Ophthalmology Residents) in the First Year (2010) of the Fifth Korea National Health and Nutrition Examination Survey. Osong, Cheongju, Korea: Korea Centers for Disease and Control and Prevention. 2011. |
| *Comment 2* | *The article found that the presence of diabetes is determined based on several factors (fasting blood glucose levels, current diabetes medications or insulin injections, physician diagnosis). A factor such as fasting blood glucose level was measured separately or only depending on any of the other factors recognized by the authors (for example, fasting blood glucose level + doctor's diagnosis, etc.), or how an independent factor? If this factor was measured as independent did individuals with high fasting glucose levels have this individuals been additional measured glycated hemoglobin or did they have a glucose tolerance test performed?* |
| Response 2 | We thank the reviewer for the opportunity to further clarify these points. In defining diabetes, fasting blood glucose levels were measured separately, while information on current diabetic mediation or insulin injection use and physician diagnosis were obtained through self-reported survey responses. The KNHANES measured glycated hemoglobin in 2010 for individuals with fasting glucose levels ≥126 mg/dL or a history of diabetes treatment. In 2011–2012, glycated hemoglobin was measured for all participants aged 10 years and older. To maintain consistency across all years, we used fasting blood glucose as the primary criterion for diabetes. Meanwhile, glucose tolerance test was not conducted. We have revised the sentences to avoid potential confusion regarding this aspect. Please see lines 140–144 in the revised manuscript.  **Line 140:** “Diabetes was determined based on a fasting blood glucose level ≥126 mg/dL, self-reported current diabetic medication or insulin injections, or a physician’s diagnosis. Hypertension was determined based on a systolic blood pressure ≥140 mmHg or a diastolic blood pressure ≥90 mmHg, self-reported current antihypertensive medication, or a physician’s diagnosis.” |

**Reviewer 2**

| *General*  *Comment* | *Reviewer #2: This is an interesting paper that suggests possible relationship between dry eye disease and IOP by analyzing data of large population. I would like to recommend some revision before further consideration.* |
| --- | --- |
|  | We deeply appreciate the constructive comments provided by the reviewer. We appreciate the reviewer’s constructive, detailed comments which helped us further improve our manuscript. Below we address your comments and list of changes that we made to our manuscript according to your reports. We believe that these modifications have strengthened the manuscript. We hope that the revised manuscript is suitable for publication in the *PLOS ONE*. |
| *Comment 1* | *1. Introduction: Lines 55-79: description regarding IOP seems too long and somewhat distracting. Please make the description more compact.* |
| Response 1 | We thank the reviewer for suggesting improvements of description regarding IOP. Based on the reviewer’s advice, we have revised the description regarding IOP in the introduction. Please see lines 50–70 in the revised manuscript.  **Line 50:** “Intraocular pressure (IOP), the fluid pressure in eyes, is maintained by the balance of aqueous humor secretion and drainage [8,9]. Dysregulation of IOP can deform the globe and cause eye disorders. Low IOP (<5 mmHg) may lead to globe contraction, choroidal detachment, and hypotony maculopathy [10], while high IOP (>21 mmHg) can damage the optic nerve and retinal ganglion cells, causing glaucomatous visual field defects [11,12].  IOP is related to lifestyle and health conditions [13,14]. It is usually higher in males [13], and increases with age [15], while smoking, alcohol consumption, obesity, diabetes, and hypertension also contribute as risk factors for elevated IOP [13-17]. Additionally, certain ocular conditions (e.g., thyroid eye disease) can affect IOP, potentially causing complications like glaucoma. Therefore, identifying and minimizing these factors are crucial [18,19].  Among ocular conditions that affect IOP, DED can influence the outflow of aqueous humor due to inflammation on ocular surface [20], however, the association between DED and IOP remains unclear. Some animal studies showed that the expression of certain prostaglandins (PGs) such as PGF2α can lower IOP in dry eyes [20,21], but only few human studies have been conducted and have reported inconsistent findings. One study found a significant inverse correlation between IOP and corneal staining score, which showed higher values in DED patients, regardless of their glaucoma status [22]. Another study showed that IOP was significantly lower in patients with a short tear break-up time (TBUT), a characteristic of DED [23]. However, a study on DED patients reported that while IOP was lower in these patients, the relationship didn’t reach statistical significance [24]. Therefore, more evidence is required to confirm the association between DED and IOP in large human samples.” |
| *Comment 2* | *2. Line 82 : Is there any reason the authors selected the data from KNHANES 2010-2012, as more recent data is available. Please describe the reason in the Discussion.* |
| Response 2 | We thank the reviewer for the opportunity to further clarify these points. While the KNHANES conducted ophthalmic examinations from 2008 to 2021, information on DED was only collected during the 2010–2012, necessitating the exclusive use of data from this period in our study. We determined that including this information in the methods section would provide greater clarity and have therefore incorporated it there. However, if you believe it would be more appropriate to include this in the limitations in discussion section, please provide your opinion on this matter. Please see lines 85–86 in the revised manuscript.  **Line 85:** “As the KNHANES provided information on DED exclusively during 2010–2012, we used the data from this period.” |
| *Comment 3* | *3. Discussion : sensation of dry eye symptoms can be association with increased systemic inflammatory level and pain modulation in the CNS. Would there be any possible relationship between the inflammation and IOP ? The authors can add some comments/.* |
| Response 3 | We thank the reviewer for suggesting improvements to clarity these points. The reviewer’s right. Previous research has reported that DED is associated with the expression of systemic inflammatory cytokines, including IL-6, TNF-α, and IL-1 [Yamaguchi, 2018]. Furthermore, it has been established that ocular surface nerve damage and irritation in dry eye patients are closely related to increased central nervous system sensitivity to pain [Galor et al., 2015]. Concurrently, the secretion of systemic inflammatory cytokines has been reported to influence aqueous humor outflow pathways, thereby affecting IOP [Huang et al., 2014]. We have incorporated these potential mechanisms into the discussion. Please see lines 309–311 and 314–316 in the revised manuscript.  **Line 309:** “Moreover, sensation of DED can be associated with elevated systemic inflammatory markers (e.g., interleukin-6, tumor necrosis factor-alpha) and pain modulation in central nervous system [45,46].”  **Line 314: “**Furthermore, systematic inflammation can influence the regulation of IOP outflow, and when it inhibits aqueous humor production, it may lead to a reduction in IOP [49].”  **Reference:** “Yamaguchi, T. (2018). Inflammatory response in dry eye. *Investigative ophthalmology & visual science*, *59*(14), DES192-DES199.  Galor, A., Levitt, R. C., Felix, E. R., Martin, E. R., & Sarantopoulos, C. D. (2015). Neuropathic ocular pain: an important yet underevaluated feature of dry eye. *Eye*, *29*(3), 301-312.  Huang, W., Chen, S., Gao, X., Yang, M., Zhang, J., Li, X., ... & Zhang, X. (2014). Inflammation-related cytokines of aqueous humor in acute primary angle-closure eyes. *Investigative ophthalmology & visual science*, *55*(2), 1088-1094.”  The following references were additionally added.  Yamaguchi, T. (2018). Inflammatory response in dry eye. *Investigative ophthalmology & visual science*, *59*(14), DES192-DES199.  Galor, A., Levitt, R. C., Felix, E. R., Martin, E. R., & Sarantopoulos, C. D. (2015). Neuropathic ocular pain: an important yet underevaluated feature of dry eye. *Eye*, *29*(3), 301-312.  Huang, W., Chen, S., Gao, X., Yang, M., Zhang, J., Li, X., ... & Zhang, X. (2014). Inflammation-related cytokines of aqueous humor in acute primary angle-closure eyes. *Investigative ophthalmology & visual science*, *55*(2), 1088-1094. |
| *Comment 4* | *4. IOP measured with GAT may be affected by corneal hysteresis. Wouldn't there be some relationship between the corneal hysteresis and corneal sensation?* |
| Response 4 | We thank the reviewer for the opportunity to correct these points. The reviewer is right. IOP measure with GAT may be affected by corneal hysteresis or sensation. Based on the reviewer’s advice, we have revised the descriptions of limitations in the revised manuscript. Please see lines 374–382 in the revised manuscript.  **Line 374:** “Fifth, KNHANES used a Goldmann applanation tonometer, a gold standard for IOP measurement. This technique, while more robust than noncontact pneumotonometry, remains partially influenced by corneal hysteresis and sensation [24]. The IOP measurements in this study were consistently obtained from the right eyes, while the DED status was not differentiated between eyes. Consequently, while we acknowledge the potential for measurement errors inherent to this technique, we cannot definitively ascertain whether these potential inaccuracies significantly impacted our findings. Further clinical studies on DED patients are necessary to confirm this effect.” |
